# Limited Role of the Apparent Diffusion Coefficient (ADC) for Tumor Grade and Overall Survival in Resectable Pancreatic Ductal Adenocarcinoma

**DOI:** 10.3390/diagnostics14060573

**Published:** 2024-03-07

**Authors:** Deniece M. Riviere, Marnix C. Maas, Lodewijk A. A. Brosens, Martijn W. J. Stommel, Cornelis J. H. M. van Laarhoven, John J. Hermans

**Affiliations:** 1 Department of Medical Imaging, Radboud University Medical Center, 6525 GA Nijmegen, The Netherlands; 2 Department of Pathology, Radboud University Medical Center, 6525 GA Nijmegen, The Netherlands; 3 Department of Pathology, University Medical Center Utrecht, 3584 CX Utrecht, The Netherlands; 4 Department of Surgery, Radboud University Medical Center, 6525 GA Nijmegen, The Netherlands

**Keywords:** ADC, diffusion, grading, MRI, pancreatic ductal adenocarcinoma

## Abstract

This study evaluated the relationship between apparent diffusion coefficient (ADC) values in pancreatic ductal adenocarcinoma (PDAC) and tumor grades based on WHO, Adsay, and Kalimuthu classifications, using whole-mount pancreatectomy specimens. If glandular formation plays a key role in the degree of diffusion restriction, diffusion-weighted imaging could facilitate non-invasive grading of PDAC. A freehand region of interest (ROI) was drawn along tumor borders on the preoperative ADC map in each tumor-containing slice. Resection specimens were retrospectively graded according to WHO, Adsay, and Kalimuthu classifications and correlated with overall survival and the 10th percentile of whole-volume ADC values. Findings from 40 patients (23 male, median age 67) showed no correlation between ADC p10 values and WHO differentiation (*p* = 0.050), Adsay grade (*p* = 0.955), or Kalimuthu patterns (*p* = 0.117). There was no association between ADC p10 and overall survival (*p* = 0.082) and other clinicopathological variables. Survival was significantly lower for poor tumor differentiation (*p* = 0.046) and non-glandular Kalimuthu patterns (*p* = 0.016) and there was a trend towards inferior survival for Adsay G3 (*p* = 0.090) after correction for age, tumor location, and stage. Preoperative ADC measurements for determining PDAC aggressiveness had limited clinical utility, as there was no correlation with histological parameters or overall survival in resectable PDAC.

## 1. Introduction

In pancreatic cancer, poor tumor differentiation is a statistically significant independent prognosticator of overall survival after resection, disease-specific survival, early recurrence, and post-recurrence survival [1,2,3,4]. Therefore, patients with poorly differentiated resectable tumors may particularly benefit from neoadjuvant therapy instead of upfront resection [5]. However, the histopathological grade is typically unknown when treatment decisions are made and, therefore, not useful for determining whether neoadjuvant therapy should be considered.

Diffusion-weighted magnetic resonance imaging (DWI) reflects changes in water mobility caused by alterations to the tissue environment, interactions with cell membranes, and macromolecules, thus providing a tissue contrast that differs from conventional T1- and T2-weighted images [6]. Generating qualitative and quantitative parametric image maps based on the calculated diffusion coefficient, the apparent diffusion coefficient (ADC) is uncomplicated. Glandular formation is the critical morphological characteristic for grading differentiation of PDAC. Neoplastic tubular and duct-like structures of well-differentiated adenocarcinoma may provide fewer structural limitations and higher ADC, while poorly differentiated ductal adenocarcinoma with limited to no glandular formation may show less diffusion due to its high cellularity. The change in tissue organization to a more solid and compact architecture may account for the restriction of diffusion of water molecules and lower ADC values [7]. If the degree of glandular formation in different grades of pancreatic cancer, indeed, plays a key role in the degree of diffusion restriction and ADC value, we might be able to identify relevant pretherapeutic high-risk patients.

Adsay et al. proposed a grading system reporting the primary and secondary patterns of glandular formation within PDAC, which demonstrated a good correlation with clinical outcome [8]. Similarly, Kalimuthu et al. found that their morphological pattern-based groups correlated better with clinical outcomes than the conventional differentiation-based World Health Organization (WHO) classification. The patterns were categorized into two components based on the presence or absence of well-formed glands [9]. While previous studies showed conflicting results regarding the relationship between ADC and WHO tumor grade of pancreatic cancer [7,10,11,12,13,14,15,16,17,18,19], no studies have investigated this relationship for other classifications.

The purpose of this study was to determine if the ADC value of pancreatic ductal adenocarcinoma could be a predictor of tumor aggressiveness and to assess its association with tumor grades according to WHO, Adsay, and Kalimuthu classifications, using whole-mount pancreatectomy specimens.

## 2. Materials and Methods

### 2.1. Patients

Our institutional review board approved this single-center retrospective study and the need to obtain informed consent was waived. Contrast-enhanced MRI with DWI has been a part of our standard diagnostic workup for patients with potentially resectable pancreatobiliary disease since January 2012. We reviewed our radiology imaging database to identify patients who underwent contrast-enhanced MRI of the upper abdomen combined with a DWI from January 2012 to December 2016. All patients aged 18 years and older with pancreatic ductal adenocarcinoma were eligible for inclusion. Patients who had undergone previous treatment for pancreatic ductal adenocarcinoma (surgery, neoadjuvant chemotherapy, radiotherapy, and ablation) were not eligible for inclusion. Special subtypes of PDAC, including colloid carcinoma, medullary carcinoma, undifferentiated carcinoma, and undifferentiated carcinoma with osteoclast-like giant cells of the pancreas, were excluded as they constitute a small subset of PDACs (1–3%) with distinct clinicopathological features. Clinical information and survival rates until 31 December 2021 were retrieved from the electronic patient files. Survival was calculated from the date of diagnosis to the date of death.

### 2.2. MRI Technique

The MR imaging examination was performed on a 3.0 Tesla system (Magnetom Skyra, Siemens Healthcare, Erlangen, Germany). Single-shot spin-echo echoplanar imaging DWI was conducted in the transverse plane with monopolar diffusion gradients along three orthogonal directions, utilizing a combination of three b-values (0/50, 400/500, and 800 s/mm^2^). ADC maps were automatically generated based on the available b-values on a voxel-by-voxel basis using the software supplied with the MR unit (Syngo VD; Siemens Healthcare, Erlangen, Germany). Additionally, axial and coronal T2-weighted sequences and axial fat-suppressed T1-weighted sequences before and after intravenous administration of gadoterate meglumine (0.5 mmol/mL; Dotarem, Guerbet, Villepinte, France) were acquired, serving as anatomical reference.

### 2.3. Image Analysis

**Figure 1:**
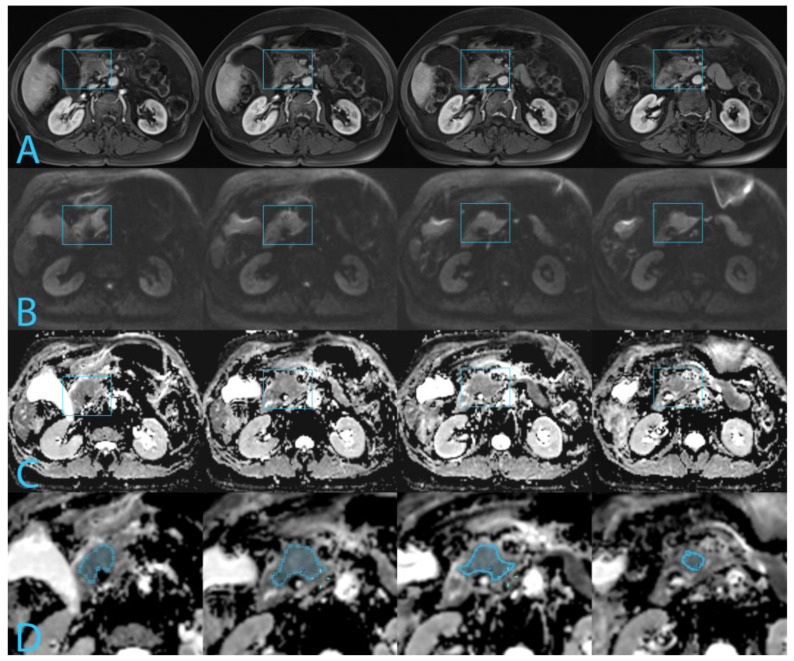
Four slices of MR images depict a pT2N2 tumor measuring 35 mm in the pancreatic head, highlighted by the blue rectangle. The tumor exhibits an ADC p10 of 1038 µm^2^/s. Histopathologically, the tumor is classified as WHO moderately differentiated, Adsay G1 and a Kalimuthu tubulopapillary pattern. (**A**). T1-VIBE arterial phase. (**B**). DWI at b800 s/mm^2^. (**C**). ADC map. (**D**). Freehand regions of interest along the border of the tumor on the ADC map.

All imaging data were retrospectively reviewed by a radiology resident with 5 years of experience, supervised by a radiologist with 20 years of experience in abdominal and pancreatic imaging. Interobserver variability is reported to be good to excellent for all MRI sequences [20]; therefore, consensus reading was deemed sufficient. Anonymized MR images were imported in MeVisLab (Bremen, Germany). The tumor was localized on the DWI using the other MR sequences (HASTE and pre- and post-contrast T1 VIBE) and contrast-enhanced CT images. Freehand regions of interest (ROIs) were drawn along the border of the tumor on the ADC map to cover the largest possible area of tumor in each tumor-containing slice. Care was taken to avoid dilated pancreatic duct, cystic lesions, or artefacts in the regions of interest, See Figure 1. ADC values for the tumor were measured with in-house developed software MeVisLab using the ROIs drawn to create a whole-volume ROI. The 10th percentile of the ADC of the whole-volume ROI was used in the analysis, assuming that tumor areas with poorest differentiation coincided with the lowest ADC.

### 2.4. Assessment of Histologic Tumor Grade

The whole-mount specimens were fixed in formalin and stained using haematoxylin and eosin. Histological examination included: grade of differentiation (World Health Organization); pTNM classification; number of lymph nodes retrieved from the specimen and number and site of lymph nodes containing metastases; and resection margins. Positive resection margins were defined as direct extension or distance of the tumor from the resection margin ≤ 1 mm [21,22].

Tumor grade was retrospectively evaluated by an expert pancreatic pathologist with 10 years of experience in evaluating pancreatic cancer specimens. Tumor grades were based on the global assessment of glandular formation, mitosis, mucin, and nuclear characteristics, and subcategorized as well, moderately, and poorly differentiated PDAC. If >95% of the tumor was composed of glands, then, it was classified as well differentiated, 50–95% as moderately differentiated, and <50% as poorly differentiated [23,24]. Additionally, the whole-mount specimen was scored according to Adsay’s grading system and Kalimuthu’s grading system.

Adsay et al. defined three patterns [8]. Pattern one was defined as well-formed tubular units with complete, easily discernible borders. Pattern two was defined as incomplete, with ill-defined borders, fusion of glands, or irregular multi-lumina formation. And pattern three was defined as non-glandular patterns, including cord-like areas, individual cell infiltration, with nested or solid (sheet-like) growth patterns. The final score is the summation of the major and minor pattern identified. Grade 1 is defined as a total score of three or less. Grade 2 is defined as a total score of four. Grade 3 is defined as a total score of five or more.

Kalimuthu et al. defined four specific morphological patterns divided in glandular (conventional and tubulopapillary) and non-glandular (squamous and composite) patterns [9]. The conventional pattern was characterized by well-differentiated glands with a tubular, stellate configuration, lined by pancreaticobiliary-type epithelium. The tubulo-papillary pattern was characterized by glands with a rounded and dilated configuration, lined by a combination of foveolar gastric-type and pancreaticobiliary-type epithelium. The squamous component was characterized by nests of large polygonal cells with squamous differentiation. The composite pattern is characterized by glands that begin to lose their integrity and cohesion, forming a spectrum of patterns including sheets, nests/islands, ribbons, cords, angulated glands, single file, or dispersing as buds and single cells and cribriforming.

### 2.5. Statistical Analysis

All data were processed using SPSS (version 27) for Windows. To find relationships between ADC values and normally distributed continuous data, Pearson’s correlation coefficient was used. For nominal data, independent *t*-tests were used. For ordinal data and non-normally distributed continuous data, Spearman was used. For survival data, Cox regression analysis was used. Median overall survival was calculated and survival curves were generated using the Kaplan–Meier method, followed by the log-rank test to assess statistical significance. Overall, *p*-values less than 0.05 were considered statistically significant.

## 3. Results

### 3.1. Patient Characteristics

We reviewed our radiology imaging database (Agfa Healthcare, Mortsel, Belgium) and identified 630 patients who underwent MR imaging of the upper abdomen. The final study population consisted of 40 patients who underwent surgery with curative intent and had a final diagnosis of PDAC; see Table 1 for demographics and pathological characteristics. Tumor stage was redefined according to the UICC 8th edition in patients previously classified according to the 7th edition. The median time interval between MRI and surgery was 27 days (range 6–44 days). Of these patients, 31 underwent pancreatoduodenectomy, 8 underwent distal pancreatectomy, and 1 underwent subtotal pancreatectomy. Postoperative systemic therapy was administered to 21 patients. Data on adjuvant therapy were missing for 4 patients. The preoperative CA19-9 level nearest to the time of surgery was used in the analysis.

### 3.2. Histopathologic Results

According to WHO grading, tumors were classified as well differentiated (*n* = 3), moderately differentiated (*n* = 25), and poorly differentiated (*n* = 12). Adsay’s grading system resulted in G1 (*n* = 22), G2 (*n* = 4), and G3 (*n* = 14). Kalimuthu’s grading scheme resulted in conventional (*n* = 16), tubulopapillary (*n* = 10), squamous (*n* = 0), and composite patterns (*n* = 14), see Table 2.

### 3.3. Correlation between ADC, Tumor Grades, and Clinicopathological Variables

There was a near-significant difference (*p* = 0.050) between the ADCs of well (mean p10 1355 µm^2^/s), moderately (mean p10 1052 µm^2^/s), and poorly differentiated tumors (mean p10 1052 µm^2^/s). The ADCs of Adsay G1 (mean p10 1081 µm^2^/s), G2 (mean p10 1046 µm^2^/s), and G3 (mean p10 1074 µm^2^/s) were not significantly different (*p* = 0.955), nor were the ADCs of Kalimuthu patterns conventional (mean p10 1068 µm^2^/s), tubulopapillary (mean p10 1183 µm^2^/s), and composite (mean p10 1006 µm^2^/s), *p* = 0.117).

**Figure 2:**
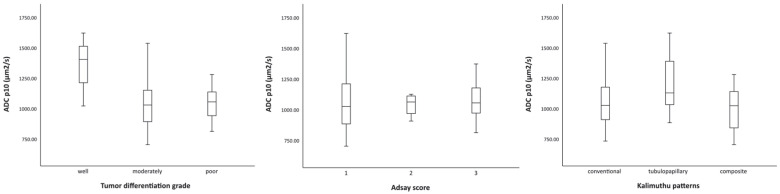
Boxplot of ADC p10 values by tumor differentiation grade, Adsay score, and Kalimuthu patterns.

There was no correlation between ADC p10 and WHO tumor grade (r = −0.119; *p* = 0.463), Adsay tumor grade (r = 0.034; *p* = 0.837), or Kalimuthu patterns (r = −0.094; *p* = 0.562); see Figure 2. ROC analysis showed population distributions almost completely overlapped; therefore, optimal cut-off values for ADC were not calculated for well/moderately vs. poorly differentiated tumors (AUC 0.488, 95% CI 0.305–0.671, *p* = 0.899), Adsay G1/G2 vs. G3 (AUC 0.475, 95%CI 0.294–0.657, *p* = 0.790), and Kalimuthu conventional/tubulopapillary vs. composite tumors (AUC 0.367, 95% CI 0.185–0.548, *p* = 0.150). ADC p10 was significantly associated with age (r = −0.316; *p* = 0.047). However, further analysis revealed this was caused by an outlier, a large duct-type pancreatic cancer with an ADC p10 1627 µm^2^/s (r = −0.149; *p* = 0.365). There was no correlation with gender (*p* = 0.503), tumor size (*p* = 0.358), tumor location (*p* = 0.054), tumor stage (*p* = 0.232), R-status (*p* = 0.643), lymph node status (*p* = 0.346), or Ca19.9 levels (*p* = 0.685). Additionally, ADC p10 was not a significant predictor for overall survival (*p* = 0.082).

### 3.4. Overall Survival

At the end of the follow-up period, 35 patients were deceased, with a maximum follow-up of more than 6 years.

The median OS for WHO tumor grades was 38.4 months (95% CI 7.6–69.3 months) for well differentiated tumors, 14.1 months (95% CI 4.2–24.0 months) for moderately differentiated tumors, and 13.3 months (95% CI 5.5–21.0 months) for poorly differentiated tumors (*p* = 0.235). For tumor grade according to Adsay, the median overall survival was 19.2 months (95% CI 0.0–40.0 months) for G1, 13.8 months (95% CI 0.0–28.6 months) for G2, and 10.6 months (95% CI 3.4–17.8 months) for G3 (*p* = 0.272). In 20 patients, Adsay’s grading system resulted in downgrading the tumor, and in four patients, it resulted in upgrading the tumor compared to the WHO classification (Table 2); however, this did not lead to an improvement in correlation with overall survival. The median overall survival for Kalimuthu patterns was 27.1 months (95% CI 0.0–56.0 months) for conventional tumors, 19.2 months (95% CI 6.0–32.4 months) for tubulopapillary tumors, and 10.6 months (95% CI 3.4–17.8 months) for composite tumors (*p* = 0.170). Overall survival was significantly lower for poor tumor differentiation (HR 0.418, 95% CI 0.178–0.985, *p* = 0.046) and non-glandular Kalimuthu patterns (HR 0.352, 95% CI 0.151–0.823, *p* = 0.016) and showed a trend for poorer survival for Adsay G3 (HR 0.498, 95% CI 0.223–1.115, *p* = 0.090) after correction for age, tumor location, and stage.

## 4. Discussion

MRI is commonly used as a diagnostic tool for suspected pancreatic cancer, particularly in cases with inconclusive findings on contrast-enhanced CT. DWI has shown promise in distinguishing benign and malignant pancreatic lesions [25], as well as detecting liver metastases [26,27] and local recurrence [28], and could be useful for assessing the response to neoadjuvant therapy [29]. However, this study revealed no significant associations between ADC p10 values of PDAC and tumor grades according to WHO, Adsay, or Kalimuthu classifications, using whole-mount specimens from surgical resections as the reference standard. PDAC ADCs did not demonstrate a correlation with different grades, showing no significant differences among low-, intermediate-, and high-grade tumors. Thus, based on our present data, it is impossible to non-invasively grade PDAC with DWI.

Previous studies have shown conflicting results regarding the relationship between the ADC and tumor differentiation, using various methods of ADC measurements, ADC values, and field strengths [7,10,11,12,13,14,15,16,17,18,19]. The variable percentage of poorly differentiated tumors across studies further suggests potential influences from differences in the study population. It is also important to highlight that in everyday clinical practice, many pathologists will use a subjective “gut-feeling approach”, relying on the degree of gland formation as the key criterion for the histological differentiation of PDAC [30]. Consistent with prior studies, our study revealed no associations between the ADC and other adverse clinicopathological features, such as tumor size, location, lymph node metastases, and R-status [16,18]. Interestingly, in pancreatic cancer liver metastases, the ADC also did not predict relevant histopathological features [31]. In agreement with Sakane et al. and Dunet et al., our study found no significant prognostic value for the ADC [10,32], while other studies found better OS in patients with tumors exhibiting high ADC values compared to those with low ADC values [14,18,19,33]. We observed a prognostic value for tumor grade, with significantly lower OS for poor tumor differentiation and non-glandular Kalimuthu patterns, and a near-significant lower OS for Adsay G3 after correction for age, tumor location, and stage. Although these three grading systems all incorporate gland formation for differentiation, it is worth noting there is not much agreement between methods.

To establish a relationship between the ADC and pancreatic cancer aggressiveness, it is critical to understand the organization of the tumor components that exist in different grades or types of tumors. The complex, dynamic, and heterogeneous tumor microenvironment of pancreatic cancer results from the cellular and extracellular components of the tumor, contributing to the inter- and intratumor variability. The predominant histopathological feature of pancreatic cancer is desmoplastic reaction, consisting of abundant fibrosis and abnormal accumulation of extracellular matrix components, which can constitute up to 90% of the tumor area. This creates a mechanical barrier and results in relatively low microvascular density [34], potentially decreasing the ADC value. Conversely, edema, small areas of necrosis, cystic parts, or large ducts have the opposite effect and tend to increase the ADC, potentially overwhelming the ADC decrease associated with cell proliferation [6].

In addition to factors related to the tumor microenvironment, technical factors such as vendors, field strength, b-values selection, and placement of region of interest [35,36] influence the ADC values. Although whole-volume measurements could, theoretically, result in higher ADCs [37], we did not observe obvious differences compared to the other studies. Moreover, whole-volume measurements better capture the morphologic intra- and intertumoral heterogeneity, characteristic for pancreatic cancer, compared to single section-based measurements [37]. Furthermore, it reduces measurement errors in the ADC values that could be introduced when a small subjective region within the morphologic heterogeneous tumor is chosen for evaluation. This is reflected in the better interobserver variability of whole-volume ADC measurements compared to solid-part ADC measurements [35,37]. Additionally, the total number of voxels used per volume of ADC value showed a great variety ranging from 34 to 1235 voxels, which is inherently related to and relative to tumor size. In prostate cancer, where the ADC is used to discriminate between low-grade and high-grade tumors, primarily in the peripheral zone [38,39], the 10th percentile ADC was the parameter that correlated best with the Gleason score and performed significantly better than the mean ADC in differentiating clinically significant cancer from clinically insignificant tumor foci. Within tumors with heterogeneous cellularity, focal areas of high cellularity are represented to a greater extent by the 10th and 25th percentile ADCs than by the mean and median ADCs [40]. Accordingly, the range of observed ADC values was the smallest for the 10th percentile.

Conducting retrospective imaging analyses is known to have its limitations. Within this study, the sample size of included patients was relatively small, resulting in the inclusion of only three well differentiated tumors, four Adsay grade 2 tumors and no Kalimuthu squamous tumors. Unfortunately, this prevents the drawing of sound conclusions regarding these subcategories. The *p*-value for the correlation between tumor ADC and overall survival initially showed proximity, but with expansion of the cohort size after the preliminary study (*n* = 10), there was a subsequent increase in the *p*-value from *p* = 0.063 to *p* = 0.082. Further enlargement of the cohort may not necessarily result in improved outcomes. Unfortunately, it was not possible to include more patients due to interference with another study. Another limitation concerned the inclusion of resected patients only. This could potentially have introduced selection bias and could confound the outcomes as these patients have a better prognosis. However, this strict inclusion criterion was also deemed a relative strength as we analyzed whole-mount resection specimens. Histopathological grade is known to more dependable in a whole-mount resection specimen whereas biopsied tissue samples can suffer from sampling bias histopathologically. Another limitation of imaging studies using DWI concerns the lack of harmonization of imaging protocols. In this specific study, the imaging protocol was different in three patients with the use of different b-values. High b-values of 800–1000 s/mm^2^ are widely used; however, the use of higher (calculated) b-values can be useful for improved delineation of PDAC because diffusion-restricted tissues show relatively higher signal intensity than the normal pancreatic parenchyma with the increasing b-values [6], thus, better capturing pure water diffusion, regardless of perfusion effects.

## 5. Conclusions

The measurement of the ADC for determining tumor aggressiveness in individual patients with resectable pancreatic cancer is not useful, as there is no correlation with histological grade or OS and there is substantial overlap in the ADC values between grades. The outcome of this study along with contradicting reports of other studies indicate there are other, yet-to-be identified factors contributing to the ADC values. To gain a better understanding of ADC values in pancreatic tumors, it might be necessary to compare in vivo MR images with whole-mount digital pathology slides to identify spatially discriminating imaging features, as has been done for prostate [41,42] and renal tumors [43].

## Figures and Tables

**Table 1:** Patient characteristics.

***Demographics***	
**Age**	median 67 years, range 36–79
**Gender**	
Male	23
Female	17
**Tumor location**	
Pancreas head	31
Pancreas body/tail	9
**Ca19.9**	median 190 kU/l, IQR 42.5–520 (missing = 8)
**Survival**	
Median overall survival	14.1 months (95% CI 11.7–17.1 months)
5-year survival	11%
***Pathological characteristics***	
**pTNM (8th edition)**	
1A	4
1B	4
2A	1
2B	11
3	19
4	1 *
**Tumor size**	median 32 mm, IQR 25–36 mm
**Lymph node metastasis (pN+)**	31
**Residual disease**	
R0	15
R1	21
R2	4
***MRI characteristics***	
**ADC**	
Mean ADC	1344 µm^2^/s (SD = 240)
Mean ADC p10	1075 µm^2^/s (SD = 209)
Mean volume	412 voxels (range 34–1235)

* Based on distant lymph node metastasis sampled during surgery.

**Table 2:** Histopathological classification.

				Kalimuthu					
	Conventional			Tubulopapillary			Composite		
				Adsay					
WHO	Grade 1	Grade 2	Grade 3	Grade 1	Grade 2	Grade 3	Grade 1	Grade 2	Grade 3
Well differentiated	1	0	0	2	0	0	0	0	0
Moderately differentiated	9	1	1	6	1	0	3	1	3
Poorly differentiated	0	1	3	0	0	1	1	0	6

There were no Kalimuthu squamous tumors identified.

## Data Availability

The data presented in this study are available on request from the corresponding author.
